# Viable cell sorting by fluidized bed centrifugation enables novel cultivation strategies

**DOI:** 10.3389/fbioe.2025.1667343

**Published:** 2025-09-16

**Authors:** Martin Saballus, Lucas Nik Reger, Robin Obser, Julia Niemann, Rene H. Wijffels, Dirk E. Martens, Markus Kampmann

**Affiliations:** ^1^ Sartorius, Corporate Research, Göttingen, Germany; ^2^ Bioprocess Engineering, Wageningen University, Wageningen, Netherlands

**Keywords:** cell sorting, fluidized bed centrifuge, process intensification, intermediate harvest, continuous biomanufacturing, monoclonal antibody

## Abstract

In biopharmaceutical manufacturing, continuous perfusion cultivation enables high space-time yields and increased plant utilization, which are critical targets for modern upstream process intensification. However, filter-based cell retention devices, utilized in these processes, have significant disadvantages: Significant sieving effects and the risk of filter blockage alongside the retention of harmful substances and non-viable cells, represent a major challenge and often reduce the viability of the culture. To enable the next-generation of continuous processes, novel cell retention strategies are required. Therefore, the aim of this study was to develop an approach for large-scale sorting of viable and non-viable cells and to investigate its applicability for novel continuous cultivation strategies. To remove non-viable cells and thus to enrich viable cells in the culture, a single-use fluidized bed centrifuge (FBC) was used, which is usually applied for concentration and washing of mammalian cells. A novel FBC method was introduced by overloading the centrifuge chambers that allows high throughput sorting depending on the culture´s viability. The impact of the sorting on the subsequent cultivation and productivity of the cells was investigated in a multi-parallel 15 mL bioreactor setup. Cell sorting after regular fed-batch cultivation showed +14% increase of viability, continued cell growth, and thus +13% higher titers. Thereafter, periodic cell sorting was tested on a 5-L scale bioreactor, combining the advantageous characteristics of fed-batch and perfusion cultivation. The feasibility was successfully demonstrated for 20 days, achieving a high average space-time yield of 0.75 g/L/d. In both cultivation trials, up to +38% higher cell specific antibody productivities were found after cell sorting. Overall, the FBC sorting method in combination with innovative cultivation concepts addresses current limitations and challenges of continuous biopharmaceutical manufacturing and has great potential to further advance modern process intensification.

## 1 Introduction

A continuously rising number of approved biopharmaceuticals, in particular monoclonal antibody (mAb) products, has driven the fast-growing bioindustry for decades (Lu et al., 2020; Walsh and Walsh, 2022; Mullard, 2021). In the past years, a notable shift in approvals from novel mAb products towards biosimilars could be seen, a trend that significantly increased the cost pressure for these products (BioPlan AssociatesInc, 2024). Consequently, efforts have been increasing to reduce development and manufacturing costs significantly. For the production of mAbs, mammalian suspension cells, such as Chinese hamster ovary cells (CHO), are predominantly used, whose costly and time-consuming cultivation represents an intrinsic bottleneck. To decrease costs and increase space-time yields, bioprocess intensification strategies have been developed targeting highly productive host cells, high cell concentrations during manufacturing, and increased plant utilization (Müller et al., 2022). For those intensified cultivation processes, maintaining the cell population in a proliferating or at least in a viable, productive state is essential.

Continuous perfusion processes are able to keep the cells proliferating and thus productive for weeks or even months using membrane-based cell retention devices to exchange the spent media against fresh media. However, in addition to the fouling risks of these membranes, such retention devices can show significant sieving effects, which results in substantial product retention and accumulation of impurities (Hayati et al., 2025; Pappenreiter et al., 2023). Moreover, frequent or continuous bleeding of cells by discharging a specific amount of cell broth is needed to provide space for the dividing cells and to remove the dead cells from the process (Pinto et al., 2020). As a result, also a fraction of still productive viable cells is removed, alongside part of the product and thus the process loses efficiency. These aspects, together with the high media consumption, and the high complexity of process control are remaining challenges for perfusion approaches.

Therefore, a large proportion of industrial biopharmaceutical manufacturing processes is still based on established fed-batch (FB) operations, where a concentrated feed is added to supply the cells with a constant nutrient level (BioPlan AssociatesInc, 2024). However, FB processes are ultimately limited, as inhibitory and cytotoxic molecules can accumulate over time or insufficient nutrient supply can occur, both of which lead to cell death. Furthermore, the apoptotic cells release host cell impurities that require elaborative downstream processing and thus reduce process efficiency. Therefore, FB processes are usually terminated after 12–15 days.

To overcome these limitations, cell sorting could be beneficial to separate the productive and proliferating cell fraction from the non-viable, impurity-releasing cell fraction. Most of the cell separation technologies are affinity-based and usually applied for biomedical sorting of different patient cell types (Dainiak et al., 2007; Bacon et al., 2020). However, there is currently no affinity marker that can be used to separate dead cells from viable cells that is also economically feasible at production-scale.

**FIGURE 1:**
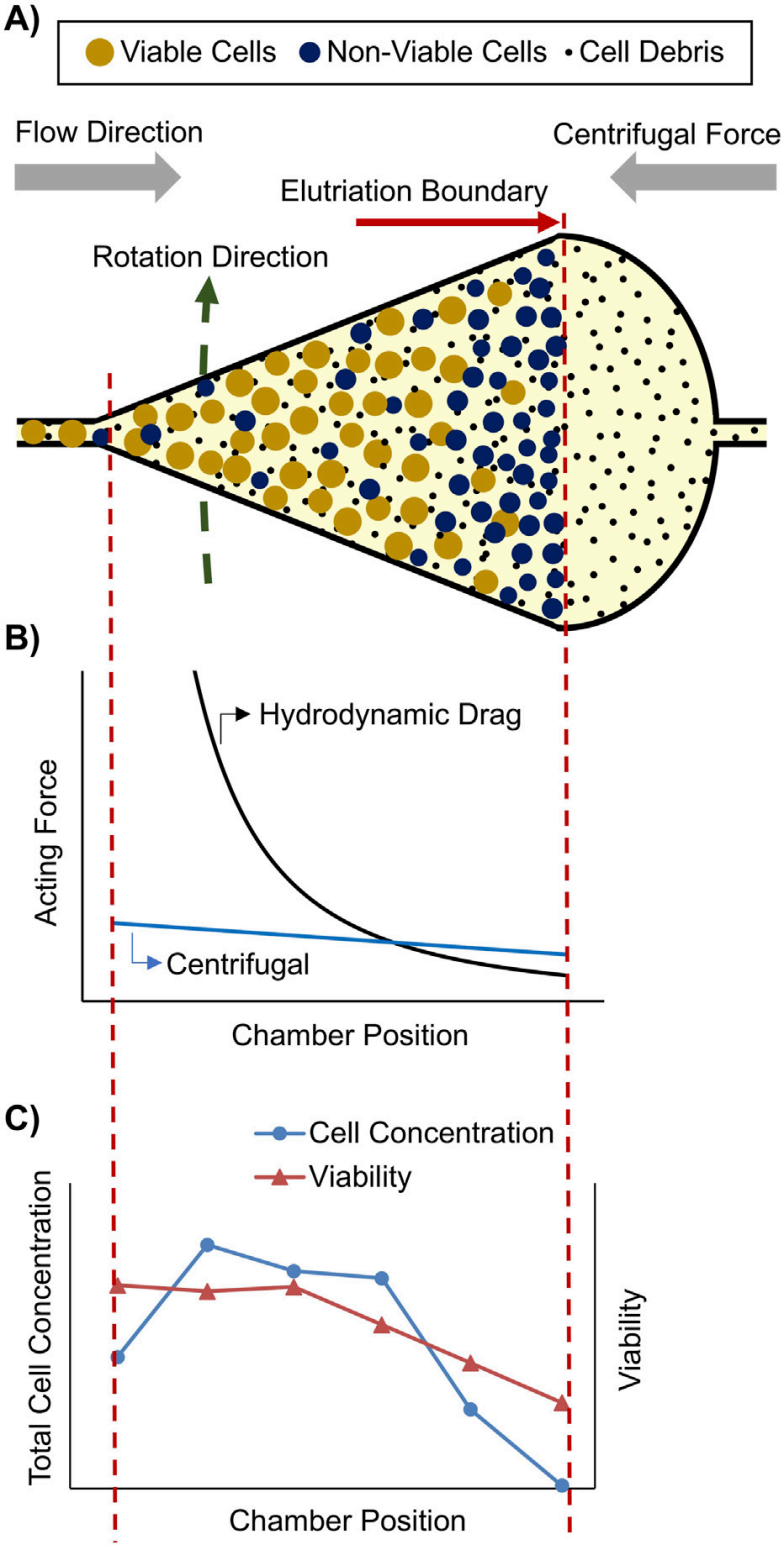
Capture and separation of a mammalian cell population inside a fluidized bed centrifuge (FBC) chamber: **(A)** Illustration of an FBC chamber completely loaded with a cell population consisting of larger and smaller cells, as well as cell debris. **(B)** Schematic hydrodynamic drag force and opposing centrifugal force inside the FBC chamber (between red dashed lines). **(C)** Experimental determined total cell concentration and viability inside a loaded FBC chamber.

An alternative sorting technology based on cell morphology is cell elutriation using a counter-flow centrifuge, also named fluidized bed centrifuge (FBC). Such systems are able to capture mammalian cells inside a fluidized bed by loading cells in a counter-current mode into a rotating chamber (Figure 1A). The cells are captured at a certain position inside the chamber at which the hydrodynamic drag force equals the counteracting centrifugal force (Figure 1B). This capture position is influenced by chamber dimension, fluid flow rate and viscosity, cell and fluid density, and cell size (Kelly et al., 2016). Due to the pear-shaped chamber dimensions, the flow velocity and thus the hydrodynamic drag force decreases significantly from the chamber inlet to the outlet, whereas the centrifugal force only decreases slightly towards the center of the rotor (Figure 1B). As all physical properties of the media and the monoclonal cells can be assumed constant, except of the cell size, the larger, mostly viable cells are enriched in the tip of the chamber and the smaller, mostly non-viable cells are enriched at the broader chamber outlet, whereas sub μm cell debris pass the elutriation boundary and are washed out of the chamber (Figure 1A).

To use FBC for cell sorting, an approach is to wash the filled chamber with a density gradient buffer, where smaller cells are elutriated from the chamber with increasing buffer density (Morijiri et al., 2013). A similar effect can be achieved by increasing the feed flow rates, which is used for blood and plasma separation since decades (Persidsky und Ling, 1982). In addition, it has been shown that the method can be used to sort cells using small morphologic differences occurring during the cell cycle (Davis et al., 2001).

However, all existing cell sorting technologies are only established at milliliter to lower liter scale to sort less than ∼ 10^9^ cells in total within a few hours (Witek et al., 2020). Therefore, they are only used for cell line development, research, and biomedical applications (Chakrabarti et al., 2024; Lu et al., 2023). To use cell sorting in industrial biopharmaceutical production a scale-up to process hundreds or even thousands of L cell broth with up to 100 x 10^6^ cells/mL is needed, which equates to 10^11^–10^12^ cells in total.

Recently, it was shown that a single-use FBC developed for washing and concentration of cells is also efficient to clarify those high cell density cultures (Saballus et al., 2021) and can achieve throughputs of more than 350 L/h processing 20 x 10^6^ cells/mL (Saballus et al., 2023). Moreover, the gentle capture of the cells in the FBC chamber allows to re-use them in a subsequent cultivation process. In a novel process scenario, called intermediate harvest (IH), the cells were concentrated, washed, and further cultivated in their exponential phase, resulting in more than 60% higher peak cell densities and more than 150% higher mAb amounts compared to a standard FB process with the same duration (Reger et al., 2023a). Based on this scenario, a highly productive continuous process with FB characteristics was developed, called continuous FB (cFB), where the cells were washed in an interval of a few days and re-cultivated (Reger et al., 2023b). Compared to continuous perfusion, the cFB achieves significant savings of culture media, but also requires a cell bleed to maintain the level of proliferating cells.

As part of the cFB studies, the cell concentration gradient and the distribution of cell viability in fully loaded FBC chambers were also investigated. It was observed that the captured viable cells were enriched in the tip of the chamber while non-viable cells were enriched at the chamber outlet close to the elutriation boundary (Figure 1C). These unexpected findings indicated that slight density and size differences between viable and non-viable cells can be used for their separation by a scalable FBC.

To take advantage from the sorting effect, the aim of this study was to develop a novel high-throughput FBC method for large-scale cell sorting. Furthermore, the feasibility of cell sorting during cultivation and its potential to intensify biomanufacturing was investigated.

## 2 Materials and methods

### 2.1 FBC operation

To develop and test a cell sorting method, two FBC system sizes were applied being the small-scale Ksep® 50 FBC system (Sartorius) with two 25 mL chambers, and the large-scale Ksep® 400 FBC system (Sartorius) with four 100 mL chambers. Appropriated cell harvest consumable kits (Sartorius) were used. Process parameters were chosen for both systems that have already been shown to be suitable for the concentration and washing of CHO suspension cells, in accordance with the results of large-scale FBC parameter optimization (Saballus et al., 2021) and used in an approach for alternating intermediate harvest of mAb (Reger et al., 2023b). In brief, the FBC´s were operated in the “cell wash harvest” mode. For each FBC system, the maximum possible centrifugal force was applied in all trials, which was 2,000 xg in the small-scale and 1,000 xg in the large-scale. Unless stated otherwise, a flow rate of 50 mL/min per chamber for small-scale and 100 mL/min per chamber for large-scale was used for cell loading and washing. For both FBC sizes, cells inside the loaded chambers were washed by exchanging 2.6x times the chamber volume with buffer in the non-sterile trials or fresh cell culture media in the sterile trials. For the FBC processes in which the processed cells were further cultivated, sterile connections of bioreactor, intermediate storage vessels, and sterile FBC consumable kits were provided utilizing BioWelder® and BioSealer® TC systems (Sartorius).

### 2.2 Cell line and medium

Two different CHO cell lines were used, both CHO-DG44, carrying the DHFR selection marker and a proprietary vector system, provided by Sartorius Stedim Cellca. Each cell line stably expresses a humanized IgG1 mAb. For the feasibility trials in FB mode, NISTmAb RM 8671, lacking a specific antigen and specified by the National Institute of Standards and Technology (NIST), was used as a well-characterized, representative mAb standard. For the FBC sorting trials in cFB mode, a mAb construct targeting the IgE antigen was expressed as an industry-relevant product. Both CHO-DG44 cell lines were cultivated within the chemically defined 4Cell®CHO media platform (Sartorius) comprising a basal and two feed media system. To diminish differences, all seed trains were conducted in similar manner to ensure equal point of origin in the production cultivations.

### 2.3 Feasibility trials in fed-batch mode

To test feasibility of the new developed cell sorting within a bioprocess an FB model process for production of a NIST mAb was set-up. In detail, a 5-L benchtop-scale bioreactor (Sartorius) was filled with 3.3 L and inoculated at 0.3 x 10^6^ cells/mL, and cultivated at 36.8 °C, DO setpoint of 40%, and pH at 7.1 which was controlled by CO_2_ gassing. After a 3 days batch phase, daily addition of 4% feed A and 0.4% feed B based on the starting volume was conducted, consecutive to day 5 a glucose feed was added to reach a concentration of 4.5 g/L in the culture. When the viability had dropped to 70%, which happened at day 12, a part of the cell broth was processed by a small-scale FBC (see section 2.1) and re-inoculated in Ambr® 15 small-scale bioreactor vessels (Sartorius) with enriched basal media. Three different approaches were conducted in triplicates: Without any processing by FBC (std. FB); with media exchange by FBC (IH), and with media exchange and cell sorting by the FBC (IH sorted). In the small-scale cultivation, similar setpoints- and proportional feeding scheme as for the 5-L bioreactor were used. The cultivation was finished after 4 days.

### 2.4 FBC sorting for continuous fed-batch

**FIGURE 2:**
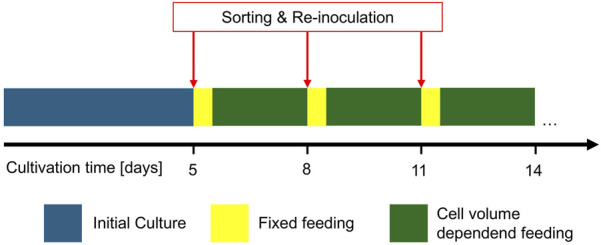
Schematic sequence of the developed continuous fed batch cultivation scenario with media exchange and cell sorting in a cyclic 3-day interval and adapted feed strategy in between.

The FBC cell sorting was further tested with a previously developed cultivation scenario, named continuous FB (cFB) as described in detail by Reger et al. (2023b). As cFB model, the scenario with a FB cycle interval of 3 days and re-inoculation with 20 x 10^6^ cells/mL was selected, producing an industrial relevant mAb. In contrast to the established cFB process, additional cell sorting was performed with each cycle interval (Figure 2). Similar to the feasibility trials (section 2.3), a 5-L benchtop-scale reactor was used and inoculated at 0.3 x 10^6^ cells/mL with 3 L initial culture volume. Similar setpoints for DO, pH and temperature as well as a similar feeding strategy up to day 4 was used. The first FBC media exchange and cell sorting was performed at day 5 using a large-scale FBC (section 2.1). 12 h after each FBC sorting, a reduced feeding scheme with 2% feed A and 0.2% feed B, relative to the inoculation volume, was performed (Reger et al., 2023b). Thereafter, a feeding scheme was applied based on the viable cell volume as described in former previous work until the next FBC processing. This cycle was repeated each 3 days until the cultivation reached a viability below 70%.

### 2.5 Analytics

The Cedex HiRes (Roche) was used to measure cell broth characteristics such as viable cell concentration (VCC), viability and cell diameter. Metabolic parameters and pH were measured with the ABL800 basic (Radiometer). All samples were centrifuged at 6,600 xg for 5 min (Centrisart A-14, Sartorius) and supernatants were analyzed.

The concentrations of mAb and the aggregation ratios were determined by size exclusion chromatography using a Vanquish™ Flex UHPLC System (Thermo Scientific) and Yarra™ 3 μm SEC-3000 columns (Phenomenex). Chromatography was performed at a flow rate of 1 mL/min running buffer (100 mM Na_2_SO_4_, 50 mM NaH_2_PO_4_, and 50 mM Na_2_HPO_4_; chemicals supplied by Carl Roth). For each mAb, separate concentration standards were measured in the range of 25 to 2,000 mg/L to determine specific calibration factors between mAb peak area and its concentration.

In order to compare different cultivation processes, the above-described measurements were used to calculate the integral of viable cell count (IVCC), integral of viable cell volume (IVCV), and cell specific productivity (Qp), which had already been used in previous work by Reger et al. (2023b) and are described in detail in the Supplementary Equations S1–S4.

The glycosylation patterns of the mAb were investigated using the ProfilerPro® Glycan Profiling Assay (PerkinElmer), as described in more detail by Saballus and Kampmann (2022).

## 3 Results and discussion

The aim of this study was to develop a cell sorting strategy that can be used for intensified biopharmaceutical production. For this purpose, the chambers of a single-use FBC system were loaded with more cells than can be captured and the breakthrough of viable and non-viable cells was investigated. Based on the results of two different FBC scales, a scalable cell sorting approach was developed. Its applicability and limitations to enrich viable CHO cells from a low viable, high cell density culture was investigated using the large scale FBC system for sorting of 2 L cell broth.

As a next step, a sorting trial with subsequent cultivation of the sorted cells was conducted to assess the impact of cell sorting on the culture characteristics and mAb productivity after regular fed-batch cultivation duration. Finally, the sorting approach was tested in a novel 5-L CHO cultivation scenario, named continuous fed-batch (cFB), for production of an industrial relevant mAb. The whole fed-batch culture was sorted every 3 days in combination with the harvest of the mAb containing supernatant. For this novel cultivation concept, the cellular and product expression characteristics were determined.

### 3.1 Development of FBC cell sorting

When using FBC systems for concentration and washing of mammalian suspension cells, the systems are usually operated at their maximum chamber loading capacity (CLC_max_) to ensure high throughput, low wash buffer consumption, and high concentration of the harvested cells. The CLC_max_ of an FBC system is defined as the highest possible total number of cells per chamber that can be captured in the fluidized bed and transferred to a harvest vessel. However, the CLC_max_ is not a fixed value as it depends on the feed cell broth characteristics, such as the size distribution of cells and the density difference between media and cells, as well as on the FBC process conditions, such as the applied centrifugal force and the flow rates (Saballus et al., 2021). Therefore, the CLC_max_ and thus the maximum volume of cell broth that can be loaded into the chamber (CV_max_) is usually visual controlled and if needed manually re-adjusted. For a known CLC_max_, the CV_max_ can be calculated as a function of the total cell concentration (TCC) in the centrifuge feed stream as described in Equation 1:

$${\text{CV}}_{\max}={\text{CLC}}_{\max}\;/\text{ TCC}$$ (1)

For better comparability of different FBC sizes and different cell broths, the relative chamber loading (rCL) was determined (Equation 2), which is the quotient of the applied chamber loading volume (CV_applied_) and the system-specific CV_max_:

$$\text{rCL}={\text{CV}}_{\text{applied}}\;/\;{\text{CV}}_{\max}$$ (2)

**FIGURE 3:**
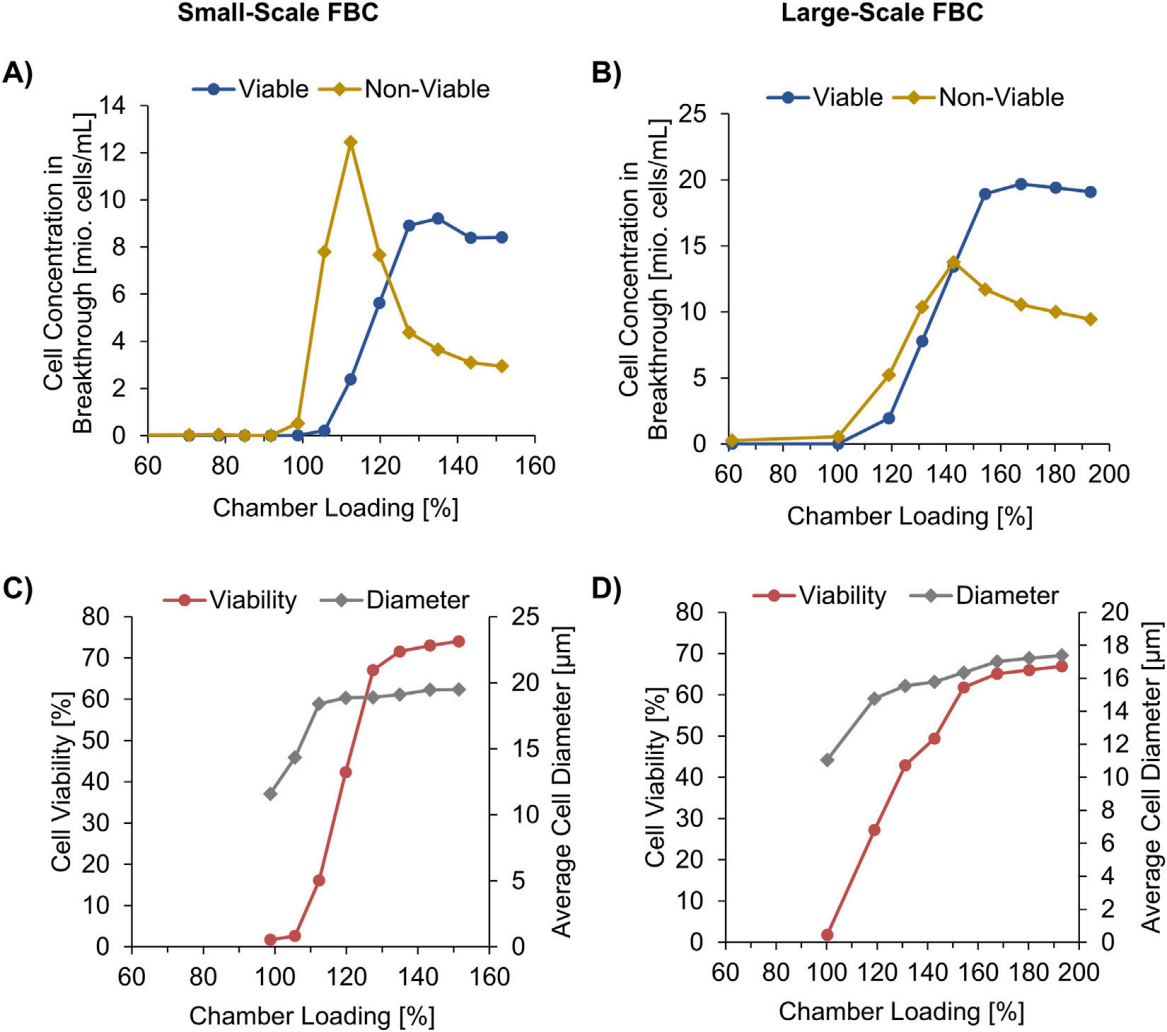
Supernatant composition of two different fluidized bed centrifuge (FBC) sizes during the chamber loading. Small-scale FBC loaded with 100 mL/min using a CHO cell broth characterized by a total cell concentration of 13.4 x 106 cells/mL, 76.5% viability, 20.2 μm cell diameter. Large-scale FBC loaded with 800 mL/min using a CHO cell broth characterized by a total cell concentration of 25.9 x 106 cells/mL total, 74.0% viability, 18.5 μm cell diameter. **(A,B)** Breakthrough of viable and non-viable cells, **(C,D)** Cell viability and average cell diameter in the collected fractions.

To investigate the separation of viable from non-viable cells inside the FBC chambers, a FB cell broth of an internal standard mAb process at harvest point was used with a CHO host TCC of 13.4 x 10^6^ cells/mL, 76.5% viability, and an average cell diameter of 20.2 µm. The chambers of a small-scale FBC with a pre-determined CLC_max_ of 4 x 10^9^ cells (not shown) were overloaded in a controlled way. During the loading, ∼45 mL fractions of the supernatant were collected and analyzed by a cell counter. Approximately 6 x 10^9^ cells in total were loaded per chamber, which corresponds to a rCL of ∼150%. Hardly any cell breakthrough was detected until CV_max_ (99% rCL) was reached (Figure 3A), which confirms the pre-determined CLC_max_. The first overloading fraction (99%–106%) contained a total CHO cell concentration of 8.0 x 10^6^ cells/mL with a very low viability of 2.6% and a low average cell diameter of 14.3 µm (Figure 3C). This confirms that the smallest cells are arranged in the chamber on the side of the elutriation boundary, as shown in Figure 1A. These cells are therefore the first to be pushed out during overloading. The results also show that the smallest cells in the culture are primarily non-viable cells. The reason for this is probably related to the morphological changes associated with apoptosis like shrinking of the cells and the release of apoptotic bodies with a diameter of 1–5 µm (Liu et al., 2018; Yu et al., 2023).

During the further overloading the concentration of viable cells, the cell diameter, and the viability ratio increased in the collected supernatant fractions, however, until 120% chamber loading the largest proportion in the fractions were non-viable cells. After the breakthrough peak of non-viable cells, the concentration of the non-viable cells decreased to an almost constant value and that of the viable cells increased to approximate the initial value. At 150% chamber loading, the cell broth composition of 11.4 x 10^6^ cells/mL TCC and 74.0% viability in the last supernatant fraction of the FBC outlet almost equaled the composition of the FBC inlet.

To develop a scalable FBC cell sorting approach, another overloading trial was conducted using a larger FBC. The main differences to the smaller FBC trial were lower maximum centrifugal force of 1,000 x g and higher applied total loading flow rate of 800 mL/min (smaller FBC: 2,000 x g, 100 mL/min). In addition, a higher maximum chamber capacity of 10 x 10^9^ cells was assumed based on (Saballus et al., 2021) due to the larger FBC chambers (smaller FBC: 4 x 10^9^ cells/Ch).

A similar sorting effect was observed that shows breakthrough of smaller, non-viable cells before the concentration of the slightly larger viable cells increased in the supernatant fractions of the overload (Figures 3C,D). Until the chambers were 100% loaded, negligible cell breakthrough was observed, similar to the smaller FBC (Figures 3A,B). In contrast to the small-scale trial, the increase of non-viable cells in the breakthrough is less sharp, which is also shown by a higher chamber overloading (>170%) applied before almost constant cell broth characteristics in the fractions were obtained. This could be caused by the combination of lower centrifugal force with higher flow rate. Another reason could be the differences in cell size distribution in the cell broth prior FBC treatment as indicated by the average diameter of 20.2 µm in the small scale trial (Figure 3C) and 18.5 µm in the large scale (Figure 3D). Nevertheless, up to the loading fraction of 131%–143% with a cell viability ratio of 49.4%, more non-viable cells than viable cells were flushed out of the chamber.

Based on the chamber loading trials with both FBC system sizes, an empirical approach for simple calculation of the chamber loading volume for efficient cell sorting (CV_sorting_) was defined (Equation 3). This approach targets scalability between different FBC sizes and a high increase in viability in combination with a high recovery of viable cells.

The function of CV_sorting_ comprises the corresponding FBC maximum chamber loading volume (CV_max_) that can be loaded into a chamber without cell loss and an additional, so far not defined, volume for chamber overloading. As indicated by the investigated breakthroughs of non-viable cells, too low overloading would lead to a low reduction of non-viable cells and thus to a low increase of cell broth viability in the harvest. A too high overloading would cause high loss of viable cells. Another variable that must also be considered is the cell culture viability in the FBC feed. The lower the viability, the more cells in total (viable and dead) must be loaded into the chamber to push out the fraction of dead cells for sufficient sorting. Consequently, the chamber loading function for efficient sorting is inversely proportional to the cell broth viability. In a process setting, the viability of the cell broth can be determined by sampling of the bioreactor before FBC sorting is started. For the FBC system and the process parameters used, the specific CV_sorting_ to be set can be calculated by dividing the corresponding CV_max_ by the measured viability:

$${\text{CV}}_{\text{sorting}}={\text{CV}}_{\max}/\text{ viability}$$ (3)

It should be noted that if sufficient cell broth is available or only a small number of sorted cells are to be re-used, larger loading volume than CV_sorting_ of the calculation approach can also be used resulting in lower cell recoveries but slightly higher sorted cell viabilities.

Similar to the calculation of the relative chamber loading (Equation 2), the relative chamber loading for cell sorting (rCL_sorting_) can be determined for better comparability:

$${\text{rCL}}_{\text{sorting}}=1\;/\text{ viability}$$ (4)

This approach was applied for all following trials.

### 3.2 Large-scale proof-of-concept

To evaluate the calculation approach for efficient cell sorting, a proof-of-concept experiment was performed to sort approximately 2 L of a CHO cell broth with a high TCC of 33.4 x 10^6^ cells/mL and a low viability of 50.7% using a large-scale FBC. In accordance with Equation 4, an rCL_sorting_ of approximately 200% was determined. Therefore, a CV_sorting_ of 588 mL was applied, taking into account a CLC_max_ of 10E9 cells and the TCC (Equations 2, 3). To ensure a full FBC cycle with the limited cell broth volume, only three of four chambers with a loading flow rate of 100 mL/min/Ch were used. Although the throughput was reduced, the entire cell broth was sorted, washed with fresh culture medium, and transferred back into the harvest vessel in less than 12 min.

**FIGURE 4:**
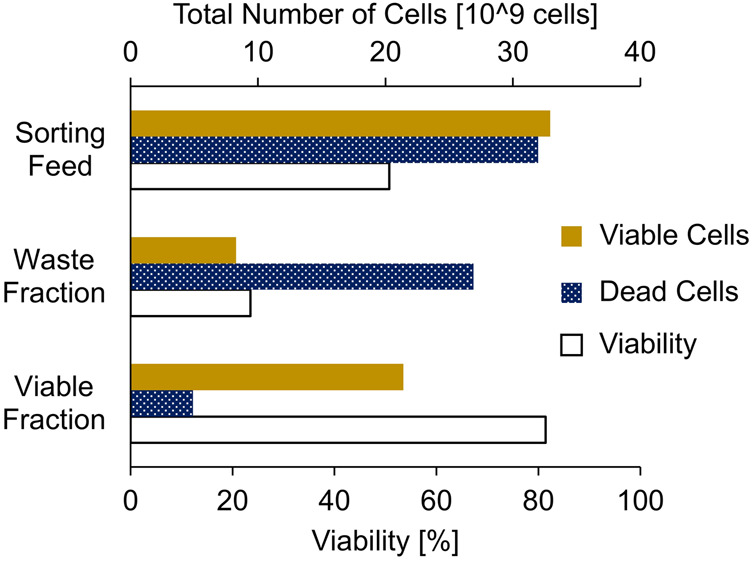
Bulk sorting of a low viable CHO cell broth feed (2.0 L; total 33.4 x 10^6^ cells/mL; 50.7% viability) by approximately 200% chamber loading of a large-scale fluidized bed centrifuge to generate a waste fraction containing most of the non-viable cells and a viable cell fraction containing most of the viable cells.

The applied cell sorting approach achieved a significant viability increase from 50.7% in the initial cell broth to 81.4% in the sorted viable cell fraction (Figure 4). Some of the viable cells were found in the waste fraction, due to the high overloading volume. However, most of the viable cells (65%) were recovered in the viable cell fraction and most of the non-viable cells (84%) were removed in the waste fraction. This demonstrated that the sorting approach is suitable for fast processing of large quantities of cells and successfully enriching viable cells in the harvest.

### 3.3 Sorting feasibility trials in fed-batch

In order to investigate the behavior of the cells and thus impact on the cultivation after FBC sorting a small-scale cultivation was conducted. To show the capabilities of the FBC sorting approach, cells were taken from the late stage (day 12) of a fed batch, with a low viability. The cell broth was split in three different approaches: A negative control without any processing (Std. FB), a control with FBC media exchange but without cell sorting (IH) similar to previous conducted work (Reger et al., 2023a), and as the third approach with FBC media exchange and cell sorting (sIH). The approaches were cultivated for an additional 4 days (day 12–16) within the small-scale system. As note, in the next passages only the 4 days (day 12–16) will be evaluated.

**FIGURE 5:**
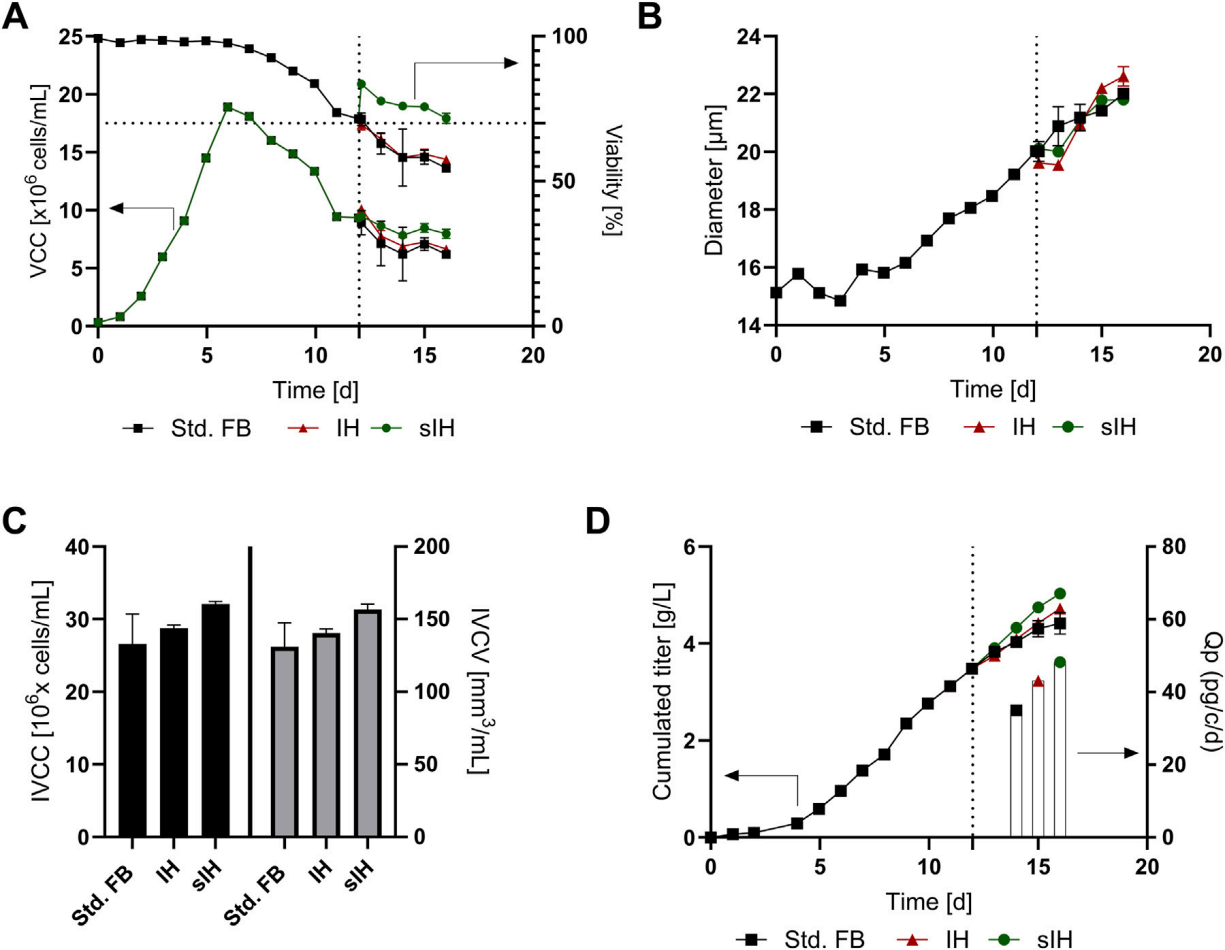
Cellular and product expression characteristics for the impact analysis of cell sorting conducted at the end of a fed-batch (FB) experiment, marked with a dotted line. Subsequent cell culture was split into three approaches: no change (Std. FB), full media exchange by FBC (intermediate harvest, IH) and the new developed sorting approach (sIH). **(A)** Viable cell concentration (VCC) and cell culture viability; **(B)** Cell diameter over the course of cultivation; **(C)** Integral of viable cell concentration (IVCC) and integral of viable cell volume (IVCV); **(D)** Cumulated titer values over the course of cultivation and cell specific productivities (Qp) for the last 4 days of cultivation.

Different performance data of the feasibility trials were determined (Figure 5). As shown in Figure 5A, only neglectable differences in the VCC between the approaches were observed after inoculation of the 15 mL bioreactors. In contrast, cell viability showed a strong increase for the sIH from 70% to 83% (+13%), which is in alignment with our previous data. This effect resulted in an elongated duration of the cultivation of 4 days (+33%) in comparison to the Std. FB and IH approaches. Furthermore, a decrease in cell diameter was visible (Figure 5B) for the two approaches processed by the FBC (IH and sIH) as expected. However, within the 4 days after the FBC processing the cell diameters increased again for both approaches and reached a similar diameter compared to the std. FB. The diameter decrease is likely caused by the rapid change in media matrix as it occurs for both cultures with media exchange. Interestingly, this effect was also visible in our previous work without cell sorting (Reger et al., 2023a). However, the cause of this effect has not yet been fully examined.

To further investigate the impact of the sorting, the integral of viable cell count (IVCC) was calculated (Figure 5C) revealing an increase of viable cells within the sIH culture (+20.7%) compared to the std. FB. To consider the previously described change of cell diameter within the process (Reger et al., 2024), the integral of viable cell volume (IVCV) was also determined showing an increase of +19.6% compared to the std. FB. The similar results for IVCC and IVCV can be explained by the observation that in the course of cultivation after sorting more cells remain viable in combination with the fact that the initially reduced cell diameter for the sIH increased again. This suggests that cell sorting has a positive effect on the overall condition of the cell population.

This effect of improved cellular state is further supported by the measured titer and cellular expression characteristics shown in Figure 5D. After transfer to the 15 mL bioreactor system, during day 12–16 of cultivation, a further increase in antibody titer can be seen for all approaches. However, the greatest increase was observed for the sIH with +1.55 g/L compared to the lowest rise in titer for the standard FB of only +0.93 g/L. To consider the differences in cell numbers, the cell-specific-productivity (Qp) was calculated for the different approaches during day 12–16. The comparison shows that the titer increase only partially results from the increased VCC but indeed is substantially influenced by a strong increase in specific productivity of +38% for the sIH compared to the std. FB. This effect is likely caused by the media exchange and FBC as visible in previous studies (Reger et al., 2023a; Reger et al., 2023b) and also for some perfusion cultivations (Gomez et al., 2020; Gagnon et al., 2018). In alignment with this hypothesis, an increased Qp was also determined for the IH, comprising only a media exchange, but not as distinctive as for the sIH approach. An explanation for this might be that the removal of a fraction of the non-viable cells by sIH leads to a lower release of inhibitory substances in the further course of cultivation, which improves the expression of the product.

Overall, a successful cultivation of the cells after FBC sorting (sIH) could be shown, resulting in higher cell numbers as well as cell viabilities post processing of the cells. In addition, the novel method leads to an elevated product expression, and thus to increased product titers at harvest. However, economic studies are necessary to evaluate whether the higher titer of only +0.62 g/L compensates for the higher costs and efforts associated with of the sIH process in a FB setting. The sIH approach probably offers more advantages if it is performed several times during a (semi-) continuous cultivation, such as an additional feature in the already developed concept of continuous FB cultivation (cFB) (Reger et al., 2023b). Therefore, the applicability of sorting in a cFB scenario was further investigated (see section 3.4).

### 3.4 FBC sorting for continuous cultivation

As outlined above, the application of FBC cell sorting in late stage fed-batch, is feasible, however, from an economic point of view, transfer of this approach towards continuous processes seems more beneficial. For a respective proof of concept study, the previously developed continuous FB (cFB) was selected (Reger et al., 2023b). In short, the cFB comprises a defined FB-cycle interval of several days per cycle, each cycle started by a complete media exchange using a FBC and cell bleed to a defined re-inoculation cell density (20 x 10^6^ cells/mL). This results in a continuous process format with superior characteristics such as low media consumption compared to a perfusion system and increased productivity compared to an FB. Our previous studies thereby showed that a starting VCC of 20 x 10^6^ cells/mL of each cycle and a FB cycle duration of 3 days was not suitable, as the proportion of non-proliferating cells in the process increased and viability decreased over the course of cultivation (Reger et al., 2023b). We anticipate that the cell sorting approach can be used to prolong cFB with a 3 days cycle duration. Therefore, this cFB scenario was selected to show the feasibility and added value of the cell sorting approach to elongate these processes and enhance the productivity.

**FIGURE 6:**
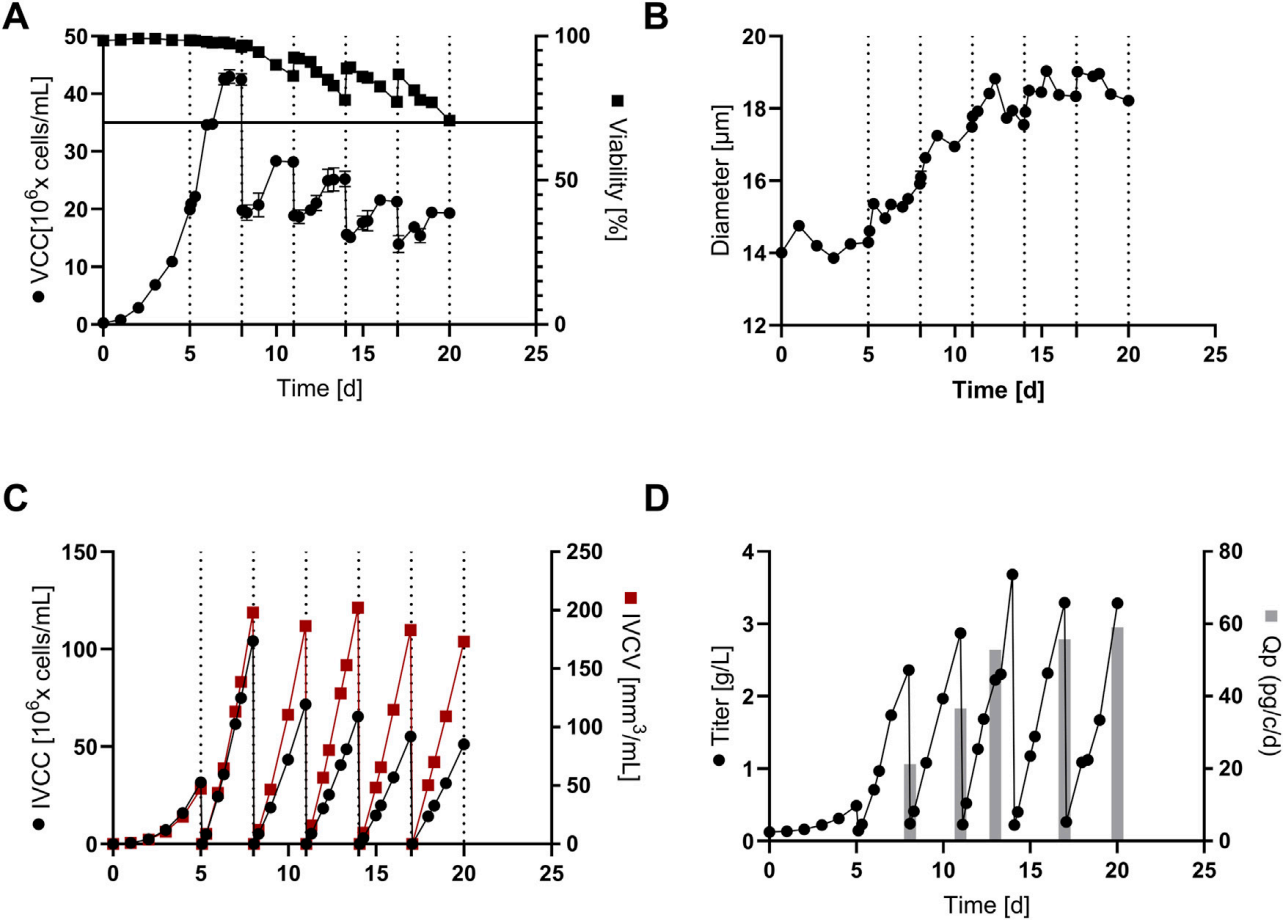
Cultivation and product expression data for the conducted continuous fed-batch (cFB) at re-inoculation density of 20 x 10^6^ cells/mL and 3 days interval of cell sorting: **(A)** Viable cell concentration (VCC) and cell viability for the process over the 20 days of cultivation; **(B)** Cell diameter; **(C)** Integral of viable cell concentration (IVCC) and integral of viable cell volume (IVCV) within the cultivation; **(D)** Antibody product titer and cellular specific productivity (Qp) for each production interval.

The cFB with cell sorting was carried out in a 5-L cultivation system and aimed at improved viability (>70%) and increased number of IH cycles compared to previous experiments without sorting. In Figure 6A the cellular performance is plotted showing overall decreasing peak cell densities in each of the FB cycles from around 43 x 10^6^ cells/mL during the first cycle to 20 x 10^6^ cells/mL during the last cycle. Similar to the VCC, the viability showed significant drops within each 3-day cycle, finally reaching the 70% marker at day 20. However, the cell sorting effect can be clearly seen at three timepoints (day 11; 14; 17) showing an increase of viability from +6% up to +12%, finally enabling a significant elongation of the process from 12 days (2 cycles) without cell sorting (historical data, see (Reger et al., 2023b)) to 20 days (5 cycles) with the novel sorting method. Furthermore, the data confirms our previous findings of a better sorting efficacy with lower cell culture viability as the increase in viability increases towards the end of the cultivation with decreasing overall cell viabilities.

In addition, a strong increase of the average cell diameter can be seen (Figure 6B), which is linked to a steady increase in the cell volume (+133%) within the cultivation. This increase in cellular diameter is consistent with previous studies within this field (Pan et al., 2017; Reger et al., 2023b) and it can be hypothesized that it is a result of a cellular ‘switch’ from cell division towards a cell size increase (Reger et al., 2024). This theory is supported by the IVCC and IVCV values visible in Figure 6C, showing a significant decrease in IVCC from 104 x 10^6^ cells/mL down to 51 x 10^6^ cells/mL for the sequence of FB-cycles. In contrast, IVCV values are relatively constant showing slight fluctuations at around 170–190 mm^3^/mL with a slight decrease in the last two FB cycles. This indicates that the overall cell volume (VCC x cell volume) that is generated during one FB cycle remains constant over the complete process duration. Interestingly, the titer values do not stabilize on a constant but show an increasing trend from around 2.3 g/L/cycle up to 3.6 g/L/cycle, with increasing values for the last 3 cycles (Figure 6D). This trend is also reflected within the Qp values increasing up to 59 pg/c/d in the last FB-cycle. In summary, around 15 g/L cumulated product titer within 20 days cultivation time were generated that equals a space time yield of 0.75 g/L/d, representing a significant increase compared to the conducted cFB process without sorting of approximately 0.54 g/L/d (historical data, see Reger et al., 2023b). Since no additional material and labor costs are assumed for the cFB process with sorting compared to the initial cFB process, it can be assumed that the increased productivity has a direct positive impact on reducing mAb production costs. To compare the cFB processes with FB and perfusion processes, however, the additional labor and material costs of cyclic FBC must be taken into account in addition to the different media consumption. For this purpose, a detailed cost of goods analysis for different production capacities should be conducted in follow-up studies.

Another aspect that should be examined in more detail is the impact of sorting on critical product quality attributes, such as aggregation, charge variants, glycosylation pattern, and functional product titer. The stability of these attributes in the various harvest pools throughout the entire cultivation process is particularly important the more sorting cycles are conducted and the longer the processing time. In an initial investigation, the glycosylation pattern of the mAb and the mAb aggregation ratio from each harvest of the process was determined, as shown in the Supplementary Material. With the exception of a slightly altered glycosylation pattern on day 11, the other harvest pools showed a consistent glycan pattern between days 8 and 20 (Supplementary Figure S1). The aggregation ratio was similar for all samples and showed a maximum of 0.84% on day 11 (Supplementary Figure S2). Compared to typical aggregation rates between 0.8% and 2.1% in FB and perfusion production processes of IgG1 and IgG4 (Walther et al., 2019), the aggregate rate of the cFB with cell sorting was at a low level. These initial results suggest that FBC processing and cyclic cultivation of cFB with sorting do not have a significant impact on glycan structures, mAb aggregate ratio, and thus the associated quality of mAb. However, further studies taking all critical quality attributes into account are required for a final assessment.

Overall, the cell sorting method was successfully applied within the cFB process mode to increase cultivation duration, without decline of the cell culture viability below the threshold of 70%. In addition, the sorting had no adverse effects on the cells and even increased their productivity. Therefore, the developed cultivation approach with integrated cell sorting represents a promising new strategy to enhance productivity in (semi-)continuous bioproduction processes.

## 4 Conclusion

In this study, a novel cell sorting approach was developed based on controlled overloading of an FBC to enrich viable cells and to remove non-viable cells from cell cultures. A calculation for the FBC parameters was found that takes into account the viability of the culture and the specific capacity of the FBC chamber, making the approach simple to adapt to different cultures and scales. For various low culture viabilities, high sorting efficiency and thus a significant increase in the viability of the harvested cells was successfully demonstrated for two different system sizes. The high FBC throughput achieved in bulk cell sorting could enable processing of complete cell cultures in biomanufacturing, whereby the cells can additionally be washed and supplied with fresh culture media.

As shown by a feasibility study, the sorting after regular FB resulted not only in an increase of viability and slower loss of viability during the continued cultivation but boosted cell specific mAb productivities. These observed positive effects on the CHO host cells can be associated with the removal of non-viable cells and inhibitory cell culture components, but this requires further investigation. Moreover, a cFB process scenario with cyclic cell sorting was developed, that also showed significant improvements in productivity and elongated cultivation duration. These results highlight the potential of FBC cell sorting to enable novel cultivation strategies that combine advantageous characteristics of continuous and discontinuous processes. In addition to its application in biopharmaceutical mAb production using CHO cells, the cell sorting approach should also be evaluated for other cell lines, such as human embryonic kidney (HEK) cells, which are typically used to produce viral vectors. Sorting could be particularly beneficial for intensifying these processes, as non-viable cells and viruses can be separated without the risk of perfusion membrane blockage. Another emerging field of applications that could benefit from FBC bulk cell sorting is found in advanced cell therapies, where the cell itself is the final drug product. Preliminary data of a follow-up study are attached to the Supplementary Material (part 3). Decreasing the burden of dead cells could not only support proliferation of live cells but increase overall quality of the cells that are returned to the patients. In summary, the cell sorting strategy developed in this study promises applicability in various scenarios and offers great advantages for the intensification of bioproduction.

## Data Availability

The raw data supporting the conclusions of this article will be made available by the authors, without undue reservation.
