# Supplementary material for: Viable cell sorting by fluidized bed centrifugation enables novel cultivation strategies
Source: Front Bioeng Biotechnol. 2025 Sep 16;13:1667343. doi: 10.3389/fbioe.2025.1667343 (PMC12481202; doi:10.3389/fbioe.2025.1667343)
Supplement: Supplementary file 1 [file Supplementaryfile1.docx]

**Supplementary Information**

This document contains the supplementary data for the research article “Viable cell sorting by fluidized bed centrifugation enables novel cultivation strategies” by Martin Saballus, Lucas Nik Reger, Robin Obser, Julia Niemann, Rene H. Wijffels, Dirk E. Martens, Markus Kampmann.

**Supplementary Part 1**

The Integral of viable cell count (IVCC) was calculated using the following Eq. 1, based on the viable cell count (VCC) at the respective time points (t).

$${IVCC}_{i}=\int_{t=0}^{t} VCC\left( t \right)dt\approx\sum_{i=1}^{n} \frac{{VCC}_{i}+{VCC}_{i-1}}{2}\times(t_{i}-t_{i-1})$$

Eq. 1: Calculation for integral of viable cell count (IVCC) over the course of cultivation

To calculate the integral of viable cell volume (IVCV), the specific cell volume (VCV) is initially required, which is calculated by multiplying the VVC by the average cell diameter (d^cell^) (Eq. 2). Based on the VCV at the respective time points (t), the IVCV can be calculated as shown in Eq. 3.

$$VCV=VCC\times\frac{4}{3}\times\pi\times\left( \frac{d^{cell}}{2} \right)^{3}$$

Eq. 2: Calculation for the specific volume (VCV)

$${IVCV}_{i}=\int_{t=0}^{t} VCV\left( t \right)dt\approx\sum_{i=1}^{n} \frac{{VCV}_{i}+{VCV}_{i-1}}{2}\times(t_{i}-t_{i-1})$$

Eq. 3: Integral of viable cell volume (IVCV) over the course of cultivation

The cell specific productivity (Qp) between the 12^th^ and 16^th^ day of cultivation for the different approaches was calculated according to Eq. 4, taking into account the mAb concentration (c) and the VCC at the respective time points (t).

$$Qp=\frac{c_{i}+c_{i-1}}{t_{i}-t_{i-1}}\times\left( \frac{{VCC}_{i}+{VCC}_{i-1}}{t_{i}-t_{i-1}} \right)^{-1}$$

Eq. 4: Calculation of the cell specific productivity (Qp)

**Supplementary Part 2**

To provide an initial assessment of the influence of cell sorting on the quality of the mAb, the glycosylation profiles of the monoclonal antibodies (Fig. S1) and the mAb aggregation ratio (Fig. S2) were added to this file. During the cyclic 3-day sorting and harvesting interval of the continuous fed-batch process, 5 harvest pools were generated between day 8 to day 20. The results showed that the glycosylation pattern between harvests remained almost the same, except for the harvest on day 11. Also the aggregation ratio was similar for all samples at a low level. Since no significant changes in glycosylation patterns and the aggregation ratio were observed between the first (day 8) and last harvest (day 20), it can be assumed that cell sorting has no influence on these quality attributes. However, further studies are necessary for a final evaluation, which should also consider other critical quality attributes such as charge variants and functional product titers.


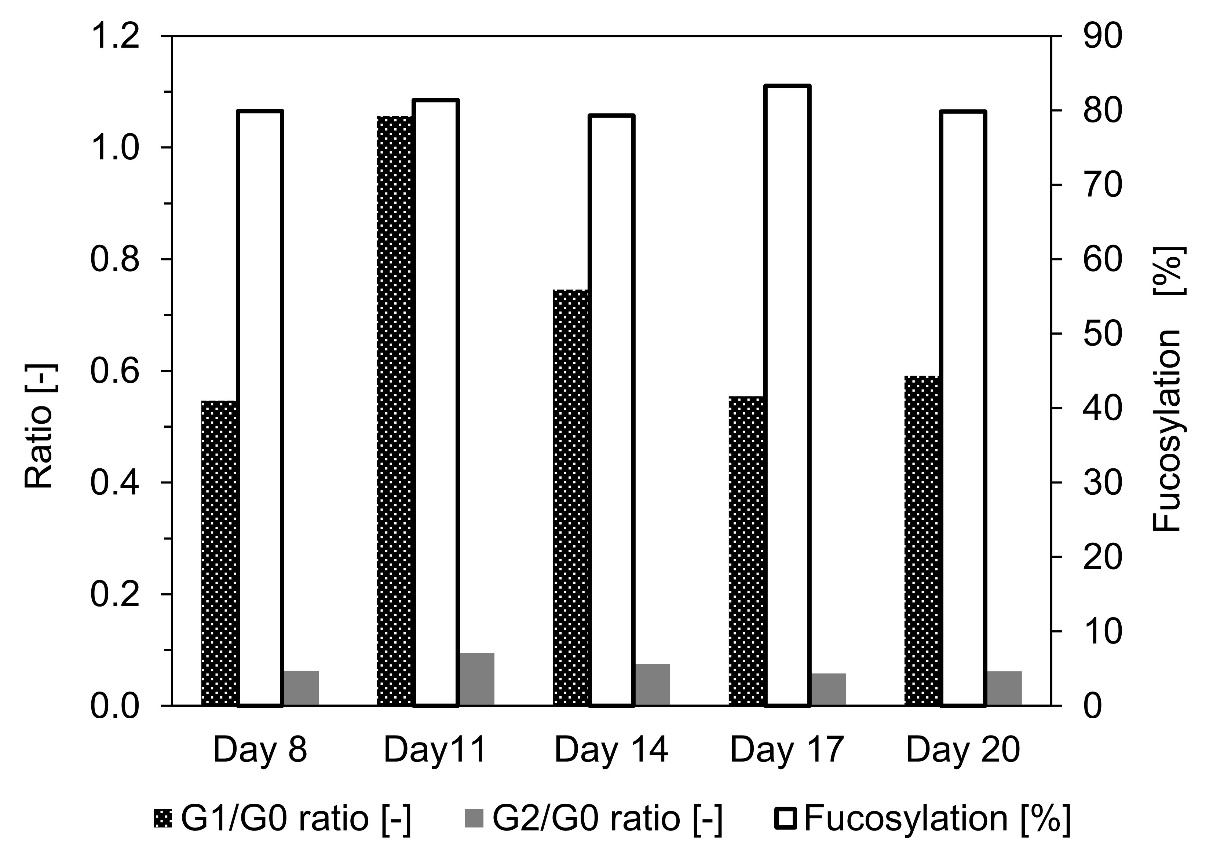


Fig. S1: Glycan profiles of the harvested monoclonal antibodies harvested on day 8 to day 20, represented by the G1/G0- and G2/G0-ratio as well as the content of fucosylated glycans in the various harvest pools of the first concept run applying the continuous fed-batch process with cell sorting (n=1).

Fig. S2: Aggregation of the mAb, represented by the ratio of the high molecular weight peak areas divided by the mAb peak area, determined by size exclusion chromatography in duplicates (n=2) for the various harvest pools (day 8 to day 20) of the first concept run applying the continuous fed-batch process with cell sorting.

**Supplementary Part 3**

In an initial concept test, a T cell suspension culture (500 mL, 39.3 x 10^6^ cells/mL) was sorted within 12 min with a fluidized bed centrifuge using a Ksep^®^ 50 FBC system (Sartorius) at 2,000 xg according to the method described in the article. Despite the already high viability of 95.7% of the culture and the significantly smaller cell diameter of 8.7 µm (tested CHO cultures between 14 µm to 20 µm), the viability of the T cells could be further increased to 97.2% through the sorting. A high recovery of 91.6% of viable T cells was achieved. These initial promising results indicate the applicability of sorting for advanced therapies as well.
